# Exploring Physicians' Dissatisfaction and Work-Related Stress: Development of the PhyDis Scale

**DOI:** 10.3389/fpsyg.2016.01238

**Published:** 2016-08-18

**Authors:** Monica Pedrazza, Sabrina Berlanda, Elena Trifiletti, Franco Bressan

**Affiliations:** ^1^Department of Human Sciences, University of VeronaVerona, Italy; ^2^Department of Economics, University of VeronaVerona, Italy

**Keywords:** physicians, job satisfaction, dissatisfaction, attachment style, scale development, mix method design

## Abstract

Research, all over the world, is starting to recognize the potential impact of physicians' dissatisfaction and burnout on their productivity, that is, on their intent to leave the job, on their work ability, on the amount of sick leave days, on their intent to continue practicing, and last but not least, on the quality of the services provided, which is an essential part of the general medical care system. It was interest of the provincial medical board's ethical committee to acquire information about physician's work-related stress and dissatisfaction. The research group was committed to define the indicators of dissatisfaction and work-related stressors. Focus groups were carried out, 21 stressful experience's indicators were identified; we developed an online questionnaire to assess the amount of perceived stress relating to each indicator at work (3070 physicians were contacted by e-mail); quantitative and qualitative data analysis were carried out. The grounded theory perspective was applied in order to assure the most reliable procedure to investigate the concepts' structure of “work-related stress.” We tested the five dimensions' model of the stressful experience with a confirmatory factor analysis: Personal Costs; Decline in Public Image and Role Uncertainty; Physician's Responsibility toward hopelessly ill Patients; Relationship with Staff and Colleagues; Bureaucracy. We split the sample according to attachment style (secure and insecure -anxious and avoidant-). Results show the complex representation of physicians' dissatisfaction at work also with references to the variable of individual difference of attachment security/insecurity. The discriminant validity of the scale was tested. The original contribution of this paper lies on the one hand in the qualitative in depth inductive analysis of physicians' dissatisfaction starting from physicians' perception, on the other hand, it represents the first attempt to analyze the physicians' dissatisfaction with reference to attachment styles, which is recognized as being a central variable of individual difference supporting caregiving practices. This study represents an original and innovative attempt to address physicians' dissatisfaction and job satisfaction. The PhyDis scale has been developed and, in line with international findings, our results indicate that role uncertainty and loss of social esteem are the most dissatisfying factors.

## Introduction

Research all over the world, is starting to recognize the potential impact of physicians' dissatisfaction and burnout on their productivity, that is on their intent to leave the job, on their work ability, on the amount of sick leave days, on their intent to continue practicing, and last but not least, on the quality of the services provided which is an essential part of the general medical care system (Dewa et al., 2014). Physicians' burnout rates range between 30 and 65% across medical specialties with particular reference to those working at the front line of clinical care, in general internal medicine and emergency medicine (Linzer et al., 2014, 2015; Schrijver, 2016). During the last decades, starting approximately about the late seventies, the practice of medicine begins to change, very rapidly, at a global level, most importantly, in all developed countries (Landon et al., 2002). Physicians, who once practiced primarily alone, are now asked to learn and adapt to work in larger groups, and begin to be subject to evaluation and approval regarding to their choice of treatments and procedures. Their autonomy shrinks sometimes consistently (Landon et al., 2002) also because of the widespread adoption of “evidence based practice” protocols and guidelines.

Economical issues, related above all to liability insurances costs, are increasingly affecting physicians' perceived job satisfaction. In fact there are different contextual factors which have contributed to the development of this last issue: the general growth of the patient-centered care, the consequent shared-decision making model, which enhances physicians engagement with patients in interactive discussions for treatment and, finally, the development of the judicialization of care (Schaad et al., 2015). Fear of crisis seems to be an important construct for organizational wellbeing (Giorgi et al., 2015) scholars demonstrate that job stress and poor social support mediate the relationship between fear of the crisis and health. In addition self-perceived poor health status, cardiovascular diseases, the development of mental illness and depression seem all to increase and to be related to the rate of unemployment (Astell-Burt and Feng, 2013; Mucci et al., 2016).

Even though hospital managers tend to shift physician-patient litigation toward conciliation, the number of complaints, regarding patients' dissatisfaction and procedural factors is exponentially increasing (Kynes et al., 2013). Moreover, a number of administrative new strategies of the health management are strongly reducing physicians' time available for deepening interpersonal relationships with patients.

Up to date literature shows that the interest toward those issues increases. A large number of in-depth analysis is available, regarding the consequences of job dissatisfaction in physicians with particular reference to burnout and its consequences: contextual ones as replacement rates and turn over intentions, those affecting the relationship with patients such as compassion, professionalism and clinical errors, and those which contribute to determine physicians' personal costs such as chronic fatigue, substance abuse, psychiatric morbidity, and suicidal ideation (Shanafelt et al., 2003; Wallace et al., 2009; Schrijver, 2016). Although, there is an increasing availability of different measures to monitor physicians' job satisfaction with their career and general wellbeing (Landon et al., 2002, 2003; Gong et al., 2014), there is a lack of instruments to assess physicians' dissatisfaction to get a 360° vision. The original contribution of this paper focuses on the priority to identify the roots of physician dissatisfaction applying a mix-method research strategy.

Therefore, aim of our research was to investigate construct and the validity of physicians' dissatisfaction (Grimm and Widaman, 2012). Moreover, since attachment style is recognized as one of the most relevant variables of individual difference affecting social competences, we assessed it in our sample in order to give a glance into the process which allows variables of individual difference to affect self-perception relating to satisfaction and dissatisfaction at work.

### Old problems and recent changes affecting negatively physicians' general work conditions

A large number of old and new problems enhance nowadays the normal burden on physicians' general conditions. Physicians and other health workers are distributed in a very uneven manner: countries with the lowest relative need have the highest number of health workers. Health care is anyway the world's largest single industry employing in excess of 59 million staff (Gottret and Schieber, 2006; WHO, 2006). In addition, even developed countries are experiencing an important shrinking of the domestic resources attributable to the welfare services. Moreover, health systems are often vulnerable during times of crisis, such as the last one, which is still ongoing. Many health systems are functioning on limited budgets and are currently understaffed. The current need for sustainable health services is increasingly dealing with issues such as the need to reduce bureaucracy, increasing cost-effectiveness, improving efficiency, decentralizing services and allocating resources to address the needs of the population (Johnson and Stoskopf, 2010). In this context, the government of the country where this study was carried out attempted during the last decade to transfer management techniques and economic/organizational models from the private sector as a basis to reorganize and “reform” public services, which are often portrayed as monolithic, inflexible, inefficient and unable to innovate. It also recently proposed to reduce significantly physicians' autonomy in their clinical practice.

Additional problems, exacerbating physicians general conditions, are related to the exponential growth of malpractice costs, related both to liability insurance and defensive medicine costs. Premium's costs for physicians are growing relating also to the increasing number of patients' complaints. Today medicine is much more complex than 50 years ago, it often requires teams instead of a single professional and very expensive drugs and technologies, moreover the exponential growth of medical knowledge is often coupled with additional relational competences which have to be reached within fields such as inter-professional team work, physicians/patient relationship, quality of care and safety (Dyrbye and Shanafelt, 2016). Patients are nowadays much more informed and therefore willing to protect and defend their right to health.

Since the economic downturn of 2008 the trend rates of complaints increased across medical specialities (Kynes et al., 2013) and across services (consumer complaints increased also during the same period from 7 to 10%). Different ergonomic and environmental conditions such as insurances' costs, health-care institutions in general, technical aspects of care and access to information, may be correlated to a number of complaint risk factors for physicians. Despite this, interesting findings are pointing at analyzing complaints by their type, including thereby physicians' variable of individual difference. Recent studies, in fact, (Kynes et al., 2013; Zengin et al., 2014; Schaad et al., 2015) agree on pointing out that the main reason which motivates patient to consult a complaints center, or to take legal action in order to restore their safety, are converging toward “the quality of interpersonal relationship with health care professionals” (Schaad et al., 2015) and communication (Kynes et al., 2013).

### Dissatisfaction scales and studies

Despite the large number of studies on physicians' job satisfaction (Lichtenstein, 1984; Cooper et al., 1989; Konrad et al., 1999; Williams et al., 1999; Lavanchy et al., 2004; Bell et al., 2006; Etchegaray et al., 2010; Mcintyre and Mcintyre, 2010; Hann et al., 2011; Mohr and Burgess, 2011; Jönsson, 2012; Bouwkamp-Memmer et al., 2013) and well-being (Firth-Cozens, 2001; Arnetz et al., 2002; Wallace and Lemaire, 2007; Janisse, 2008; Fang et al., 2011; Hills et al., 2012; Rockey, 2012; Alsuwaida et al., 2013; Bell, 2013; Aalto et al., 2014; Heponiemi et al., 2014; Simon and Durand-Bush, 2014; Min et al., 2015; Scheepers et al., 2015; Tucker et al., 2015), there is apparently little interest in physicians' dissatisfaction and its roots.

During the early'80 physicians dissatisfaction gained social and academic attention. The first study was carried out in 1984 (Lichtenstein). Even though this first attempt allowed researcher to develop an instrument for physicians' satisfaction and dissatisfaction which was found to have both convergent and discriminant validity it is totally out of date. Previous studies (Nathanson and Becker, 1973; Lichtenstein, 1984) don't help us to understand the multifaceted and complex nature of physicians dissatisfaction because researchers used either a single overall question or multiple question derived from literature, that simply were summed and reported as a single general level of physicians dissatisfaction. Various job satisfaction scales, including single-item, and multi-item scales have been employed during the last decades. The 10-item mono-factorial scale (Hills et al., 2012) used to assess job satisfaction in Europe, UK, North America and Australasia does not allow an in-depth, multifaceted approach to physician's dissatisfaction, thus preventing the differentiation between intrinsic and extrinsic factors.

The first systematic longitudinal survey on physicians' dissatisfaction was carried out in the US in the period from 1996 to 2001 (Landon et al., 2003). Findings demonstrate that physicians' dissatisfaction ranges did not change dramatically. Major driving forces of dissatisfaction are those related to threat of physicians' autonomy, to their ability to manage their day-today patient interactions and to their ability to provide high-quality care.

A recent study on dissatisfaction included a comparative assessment of results on Indian physicians' dissatisfaction with those of developed countries (Sharma et al., 2014). Dissatisfaction magnitude and its causes are similar, working environment and rates of pay were identified as major satisfaction/dissatisfaction factors. Work-overload and deprivation of autonomy associated to management outcome/ efficacy and efficiency evaluations are also reported as important dissatisfaction's factors. A study in Europe (Janus et al., 2008) is congruent with those findings. According to the AA. *non-monetary factors* are important determinant of physicians dissatisfaction, even more than *monetary incentives*. Seven principal factors were identified: decision making and social recognition, continuous education and job security and the increasing amount of administrative tasks.

The above-mentioned studies were carried out using measures mainly based on previous studies and on literature; their approach is therefore quantitative and focused on confirming previous results and already tested frameworks of reference. Because of the global need for enhancing integration of health systems all over the world (Lister and Labonté, 2009) and the widespread lack of economic resources, health systems constantly undergo a large number of changes. In addition research so far, largely failed to analyze the potential impact of different service systems within and between countries on physicians' well-being/dissatisfaction and performance (Linzer et al., 2002; Siegrist et al., 2010) We assume therefore there is an urgent need of accurate data on physicians' dissatisfaction addressing a 360° view of their perception relating to individual, environmental, and social causes.

### Consequences of dissatisfaction

According to the 2015 Medscape Physician Compensation Report physicians' dissatisfaction is increasing. Results of several studies (Reschovsky et al., 1998; Barnard and Tong, 2000; Landon et al., 2006) show that dissatisfied physicians are 2–3 times more likely to leave medicine than satisfied physicians. AA. demonstrate that dissatisfied physicians are also more likely to retire and to take that step at an early age. Women physician during their childbearing years and high-paying specialties, such as surgery, compared with lower-paid specialties, such as internal medicine, are more likely to reduce their clinical practice or retire. These finding account for a lack of relationship of this decision with income. Previous research demonstrate that unhappy physicians provide lower quality care (Linn et al., 1985; Grembowsky et al., 2005) and are unable to cope with increased physical demands and stress related to surgical specialties. Physicians' job satisfaction affects positively patients' adherence to treatment and different actions in managing their disease (Williams and Skinner, 2003) in fact dissatisfied physician have a riskier prescribing profile, less satisfied patients, less adherent patients and thus their global performance drops to a reduced quality of care (Wallace et al., 2009). In addition physicians' job satisfaction is related to patients' satisfaction rates (Haas et al., 2000; Williams and Skinner, 2003; Stelfox et al., 2005). Comparative analysis (Siegrist et al., 2010) of physicians' dissatisfaction between countries, ruled by different health-care provision systems, highlights that the highest levels of work stress is reported by physicians in Germany and the lowest level by physicians in the UK, with US physicians reporting intermediate levels. Differences are largely due to the reward component. Moreover, lower levels of administrative and clinical autonomy are associated with higher work stress in all countries. A wide range of factors may potentially raise physicians' risks of work stress (Dyrbye and Shanafelt, 2016) and dissatisfaction: frequent organizational changes, worldwide lasting crisis and subsequent lack of resources, the health system management's need for stringent outcome evaluation, increasing frequency of patients complaints, the strengthening of parameters to assess services' quality (Lister and Labonté, 2009), the growing amount of evidence based strict protocols implementation and the increasing lack of professional autonomy. Long-term exposure to stressful experiences at work and lasting job dissatisfaction can often result in burnout. Scholars (Dewa et al., 2014) show that both burnout dimensions, that is emotional exhaustion and depersonalization, are significantly associated with more use of sick leave days and with the intention to leave a current position. Moreover, physicians who have high scores for both EE and DP dimensions of burnout incur significantly greater odds of having self-perceived “insufficient” work ability (Ruitenburg et al., 2012).

## Materials and methods

Ethical approval was obtained from the ethics committee of the provincial medical board's (*Ordine dei Medici Chirurghi e degli Odontoiatri della Provincia di Verona*). The study was carried out between November 2013 and January 2015. Informed consent was obtained from each participant, after having explained the nature and purpose of the study. Participation was entirely voluntary; participants were informed about their right to withdraw from the study at any time without incurring any penalties. The anonymity and confidentiality of answers were guaranteed. Questionnaire included an information sheet that briefly described the study's purpose and a consent form.

The research was articulated into two studies: instrument development (item generation and content validity) and test of psychometric properties. The latter examined the construct validity (factor analysis and reliability) and the discriminant validity of the PhyDis scale (Physicians' Dissatisfaction Scale), that is, its correlation with the Global Job Satisfaction dimension of the Physicians' Job Satisfaction Scale (Konrad et al., 1999; Williams et al., 1999).

### Study 1: development of the PhyDis scale (physicians' dissatisfaction scale)

#### Item generation

The items for the PhyDis scale were generated through 4 focus group including 35 physicians, representatives of all specialties. Focus groups were low structured and their participants underwent a theoretical sampling procedure (Strauss and Corbin, 2008; Pedrazza and Berlanda, 2014). Participants represented both genders, and were selected having more than 10 years of clinical experience.

Participants were asked to list all potential sources of physicians' dissatisfaction. Participants were therefore free to express whatever they could perceive as root of their unease and dissatisfaction at work. They were encouraged to generate as many statements as possible. The transcripts of the focus groups were submitted to a content analysis (with NVivo 8). The qualitative analysis was structured around three conceptually progressive coding operations: open coding, axial coding and selective coding. Reliability in this type of research is construed as the degree of consistency with which cases are assigned to the same category by different observers or by the same observer on different occasions (Hammersley, 1992). Categories were used consistently. Units of meaning were included in the categories on the basis of decisions made by four different judges, who worked independently and compared their results only when they had finished their work, in order to exclude the possibility of their influencing each other. The final comparison process brought to the final issues' number through a continuous and generative re-interpretation, toward agreement- process between judges (Strauss and Corbin, 2008; Pedrazza and Berlanda, 2014). The open coding (Strauss and Corbin, 2008) allowed us to identify 276 responses. This initial set of statements was refined by removing redundant answers. Axial coding defined as a set of procedures whereby data are put back together in new ways, by making connections between categories (Kelle, 2005; Strauss and Corbin, 2008), brought researchers to identify 21 issues. A content validity index (CVI; Polit and Beck, 2006) was computed by considering the percentage of items deemed to be relevant. A CVI of 0.91 was obtained, which indicated an acceptable agreement among the experts about the validity of the measure.

The final qualitative exploration of data, performed through selective coding, enabled us to gather those items in 5 distinct dissatisfaction's areas: economical issues; lost of social recognition and public image; relationship with staff and colleagues; patient/physician relationship and physician's responsibility; and bureaucracy. *Personal Costs*: includes items related to social security and liability insurance costs. *Role Uncertainty*: this factor is associated to the following issues: negative feelings caused by the health systems' frequent negative media exposure, dealing with confronting and stressful patient's complaints, the ongoing decline in public image because of the lost of status physicians were used to enjoy. *Relationship with colleagues and staff* is referred to poor communication with colleagues and staff, rudeness, delays and inattentiveness as also the physicians' often perceived professional solitude. *Responsibility toward patients*, this factor includes items referring to patients' and their families' anxiety and worry, to discomfort in discussing and communicating a poor prognosis associated to the responsibility physicians feel to provide safe and effective care. *Bureaucracy*: Health organizations as also medical facilities are nowadays increasingly heavy in procedural documentation, in addition the high documentation demand (e.g., computer-based patient record CPR, Rahimi et al., 2008) is associated with high caseload. We used Krippendorff's alpha (Geertzen, 2012; Krippendorff, 2012) to assess the inter-rater agreement achieved among the four judges in the selective coding (α = 0.779).

At this stage we proceeded to the development of a questionnaire, about all potential sources of physicians' dissatisfaction gathered from axial coding (21 items, see Table 1), to investigate more deeply the structure and mutual relation of the five identified factors.

**Table 1:** **Item loadings of confirmatory factor analysis (CFA) with 20 items (*N* = 853)**.

**Items**	**Personal costs**	**Bureaucracy**	**Responsibility toward patients**	**Role uncertainty**	**Relationship with colleagues and staff**
Item 1. Insurance's costs for occupational accidents and diseases	0.85				
Item 2. Personal costs for contribution for personal retirement plan	0.71				
**Personal Costs**	**–**	**0.50**	**0.31**	**0.49**	**0.39**
Item 3. Relationships with medical supervisors		0.70			
Item 4. Relationship with the administrative staff		0.64			
Item 5. Increasing amount of administrative and bureaucratic procedures		0.56			
Item 6. Difficulties in Understanding the Underlying Politics of Health Care Policy Decision Making		0.48			
Item 7. Patients' Computerized medical records		0.41			
**Bureaucracy**		**–**	**0.54**	**0.67**	**0.67**
Item 8. Communication with patient's relatives and family			0.64		
Item 9 Patients' fear and anxiety			0.61		
Item 10 Responsibility toward patients			0.61		
Item 11 Communicating about a poor prognosis			0.49		
**Responsibility toward patients**			**–**	**0.82**	**0.81**
Item 12 Fear of Medical Malpractice Claims				0.60	
Item 13 Patients' Use of the Internet for Medical Information				0.60	
Item 14 General loss of social and public esteem				0.53	
Item 15 Patients making claims (litigious patients)				0.52	
Item 16 Negative media exposure of medical malpractice claims				0.42	
**Role Uncertainty**				**–**	**0.74**
Item 17 Solitude and isolation at work					0.65
Item 18 Relational problems and communication issues with colleagues					0.61
Item 19 Lack of support from medical professionals associations					0.52
Item 20 Relationships with the nursing staff					0.45
**Relationship with Colleagues and staff**					**–**

*All loadings were significant, p < 0.001. Bold value indicates the standardized relationship among ξ (ksi -Latent Variables)*.

In addition, given that the largest number of studies on physicians' dissatisfaction refer the importance of relational issues regarding both colleagues, teams and patients, we decided to include a self-report attachment style measure.

According to literature (Mikulincer and Shaver, 2007) individuals' attachment style is relevant within the present study for at least 4 reasons: (1) it represents the most important variable involved in the occurrence of relationships' issues, it supports and regulates intra and interpersonal psychological processes, which are needed in order to manage dyadic relationships regarding to any social domain: familial, social, peers' and professional's domain; (2) up to date literature states that attachment is a variable of individual difference which affects a consistent number of organizational processes (Declercq and Willemsen, 2006; Mikulincer and Shaver, 2007; Richards and Schat, 2011; Falvo et al., 2012; Pedrazza and Boccato, 2012); (3) furthermore (Mikulincer and Shaver, 2007; Adshead, 2010) care-giving or care-eliciting relationships are attachment relationships, where secure subjects are able to recognize others' need, providing responsive answers and care; avoidant subjects tend to avoid negative emotion and provide a rather dismissive caregiving and anxious subjects engage often in a rather compulsive caregiving (Dozier and Tyrrell, 1998; Leiper and Casares, 2000). Insecure attachment style is likely to be one of several individual risk factors that affect professionals' psychological well-being in helping professions and thereby it affects professionals ability to cope with threatening events at work and relational issues with patients; (4) finally attachment style affects the individual's ability to cope with threatening events of any type. Secure attached people, unlike insecure ones, are more likely to perceive as less threatening any type of negative event such as long-lasting stress, death of relatives, poor prognosis, job loss, and severe economical issues. Despite the evidence of the importance of attachment style in shaping the manner professionals manage their relationships with clients, patients, and users (Dozier and Tyrrell, 1998), only one study assessed attachment style in a convenience sample of medical students (Ciechanowski et al., 2004).

### Study 2: psychometric test

The PhyDis scale consisted of 21 items. Participants were asked to indicate to which extent each item indicates a perceived subjective source of dissatisfaction. Each item was answered on a 6-point Likert scale, ranging from 1 (not at all) to 6 (extremely). On line questionnaire reached 3070 physicians of the province. The study was presented as research on physicians' dissatisfaction.

The survey was administered to all physicians of the Verona province in Northern Italy. Three thousand and seventy physicians were contacted by e-mail. A total of 1251 questionnaires were completed, with a response rate of 40.75%. We eliminated 398 questionnaires because they had more than 20% of missing responses in the PhyDis Scale and in the Global Job Satisfaction Scale (311 questionnaires), and/or they had all missing answers in the Global Job Satisfaction Scale (88 questionnaires). The final sample consisted of 853 physicians. The gender distribution was 510 males (59.8%) and 323 females (37.9%); 20 participants have not indicated the gender (2.3%). The mean age was 51.38 years (*SD* = 11.71; *range* = 24–83; 120 missing data, 14.07%), and the mean length of service was 17.94 years (*SD* = 12.20; *range* = 0–55; 101 missing data, 11.84%).

#### Measures

The instrument included the so developed PhyDis Scale, questions on demographic and occupational characteristics, the Global Job Satisfaction dimension of the Physicians' Job Satisfaction Scale (Konrad et al., 1999; Williams et al., 1999) in order to test external validity of the proposed new scale, and Adult Attachment Types (Hazan and Shaver, 1987, 1990).

*Global Job Satisfaction* (Konrad et al., 1999; Williams et al., 1999). This dimension refers to a factor of the validated Physicians' Job Satisfaction Scale. It includes 5 items (e.g., Overall I am satisfied in my current practice) regarding pleasure with work practices, perceived level of general satisfaction at work, level of perceived frustration in the clinical practice (reverse item), the extent to which work practices meet physicians' expectations, the consistency in time of their career choice (the extent to which they would choose again their job). Responses were given on a 6-point Likert scale ranging from 1 (complete disagreement) to 6 (complete agreement). The reliability of the scale in the present study was α = 0.80.

*Adult Attachment Types* (Hazan and Shaver, 1987, 1990). This measure of attachment type offered physicians three answer alternatives (the avoidant type, the anxious type, and the secure type), of which they were to choose the one that best described how they typically felt in relationships.

#### Data analysis

All of the data were analyzed using the IBM SPSS Statistics 21.0 and LISREL 8.8 (Jöreskog and Sörbom, 2006). PRELIS (LISREL 8.7) was used for the imputation of missing data with the Expectation-maximization (EM) algorithm. Following Schafer and Graham's (2002) recommendations, maximum likelihood imputation (EM algorithm) was used to estimate values for missing scores. Maximum likelihood procedures provide more accurate estimates of population parameters than list-wise deletion or mean substitution (Schafer and Graham, 2002; see also Pedrazza et al., 2015). The validity of the PhyDis scale was tested by applying CFA (LISREL 8.8). A five-factor model was tested, in which observed variables corresponded to the 21 items and latent variables to the five dimensions identified in the qualitative analysis. The first loading of each factor was fixed to one for model identification. The goodness of fit was evaluated using the χ^2^ test, the comparative fit index (CFI), the root mean square error of approximation (RMSEA), and the standardized root mean square residual (SRMR). The fit of a model can be considered acceptable when χ^2^ is non-significant, CFI is ≥0.95, RMSEA is ≤ 0.06, and SRMR is ≤ 0.08 (Hu and Bentler, 1999). The Shapiro-Wilk test revealed that responses to the 21 items were not normally distributed (*p*s < 0.001). Therefore, robust maximum likelihood (RML) estimation was used. Compared to other estimators, such as generalized least squares (GLS) or weighted least squares (WLS), RML generally performs better under non-normality conditions in large models (Boomsma and Hoogland, 2001). The chi-square difference test (Satorra and Bentler, 2001) was applied to correlations ≥0.80 to test whether highly correlated factors were distinct from each other. For each of these correlations, the five-factor model was compared with a nested model, in which the correlation between the two latent variables was fixed to 1 (i.e., the perfect correlation) and the two factors were constrained to have equal correlations with the remaining factors (see Miyake et al., 2000; Trifiletti et al., 2009; for a different method see Trifiletti et al., 2007). Subsequently, the five factor model was compared to a one-factor solution, in which all the items loaded onto one latent variable. The two models were compared using the Akaike information criterion (AIC). Values of AIC closer to zero indicate a better model fit (Hair et al., 1998).

As a measure of reliability, the internal consistency was examined by computing Cronbach's alphas (SPSS 21.0) for the five dimensions. Reliability is generally considered satisfactory when alpha is ≥0.70 (Kline, 2000), however many authors consider an alpha values between 0.60 and 0.70 acceptable (Loewenthal, 2004). Descriptive statistics were obtained with SPSS 21.0. For each variable, a composite score was computed by averaging the respective items. Pearson correlation (SPSS 21.0) was used to examine the relationship between the five PhyDis subscales and Global Job Satisfaction. Finally, multiple linear regression analyses were conducted.

## Results

### Factor structure, reliability, and validity of PhyDis scale

The five-factor model with 21 items showed an acceptable fit: [$\chi_{\left(179\right)}^{2}$ = 789.36, *p* ≅ 0.00; CFI = 0.99; RMSEA = 0.03; SRMR = 0.05]. Although, the χ^2^ was significant, all the other indices met the respective criterion. However, the inspection of loadings revealed that one item of the factor Responsibility toward patients (“Patients' suffering and pain”) showed a low loading (0.30, completely standardized coefficient). After dropping it, the fit indices for the model were: [$\chi_{\left(160\right)}^{2}$ = 683.79, *p* ≅ 0.00; CFI = 0.99; RMSEA = 0.03; SRMR = 0.05]. The loadings were all significant (see Table 1). Correlations between the five factors (Table 2) were moderate to large (range = 0.31–0.82). The chi-square difference test showed that the correlations >0.80 were significantly different from 1 (i.e., the latent construct were distinct factors): all [$\chi_{d\left(4\right)}^{2}$ ≥ 58.37, *p*s ≤ 0.00]. The one-factor model showed an acceptable fit: [$\chi_{\left(170\right)}^{2}$ = 1318.90, *p* ≅ 0.00; CFI = 0.96; RMSEA = 0.05; SRMR = 0.07], although worse than the five-factor model. AIC values confirmed that the five-factor solution (AIC = 369.30) was preferable compared to the one-factor model (AIC = 639.88).

**Table 2:** **Descriptive statistics and intercorrelations among variables**.

		**N**	**Mean**	**SD**	**1**	**2**	**3**	**4**	**5**	**6**	**7**
1	Personal costs	853	3.78	1.47	–						
2	Bureaucracy	853	4.36	0.95	0.344^***^	–					
3	Responsibility toward patients	853	3.37	1.03	0.221^***^	0.539^***^	–				
4	Role uncertainty	853	3.30	1.03	0.302^***^	0.489^***^	0.541^***^	–			
5	Relationship with colleagues and staff	853	4.07	0.96	0.380^***^	0.473^***^	0.369^***^	0.474^***^	–		
6	Global Job satisfaction	853	4.48	1.12	−0.233^***^	−0.208^***^	−0.165^***^	−0.269^***^	−0.263^***^	–	
7	Age	733	51.38	11.71	−0.183^***^	−0.086^*^	−0.062	−0.049	0.107^**^	0.147^***^	–
8	Length of service	752	17.94	12.20	−0.225^***^	−0.105^**^	−0.039	−0.056	0.084^*^	0.092^*^	0.802^***^

* *p < 0.05*,

** *p < 0.01*,

*** *p < 0.001*.

The reliability of each dimension was examined by computing Cronbach's alphas for the five dimensions. Cronbach's alphas were 0.75 for Personal Costs, 0.69 for Bureaucracy, 0.68 for Physician's responsibility toward hopelessly ill patients, 0.67 for Decline in Public Image and Role Uncertainty, and 0.63 for Relationship with colleagues and staff.

### Descriptive statistics and correlations

The means, standard deviations, and correlations of study variables are presented in Table 2. As shown in the table, the Role Uncertainty was rated as the most dissatisfying factor, Bureaucracy immediately following. Relationship with colleagues and staff was the aspect that generated less dissatisfaction.

The correlations reveal that the five sources of dissatisfaction were positively related. Global Job Satisfaction was negatively correlated with each of the five identified sources of dissatisfaction. These results support that the two sets of measures are discriminated from each other.

Age and Length of Service were positively correlated with Global Job Satisfaction and with the dissatisfaction due to the Bureaucracy. Moreover, Age and Length of Service were negatively correlated with dissatisfaction about Personal Costs and Role Uncertainty.

### The roles of gender, age, and attachment style

There were few sex differences (Table 3). Women reported higher levels of dissatisfaction than men, with specific reference to decline in public image and role uncertainty (*p* = 0.001), physician's responsibility toward patients (*p* < 0.001); and relationship with colleagues and staff (*p* = 0.002); and on average, women were less satisfied (*p* = 0.041). There weren't differences regarding Personal Costs, and Bureaucracy.

**Table 3:** **Differences in the sample means**.

		**Men *N* = 510**	**Women *N* = 323**	***t***	**Younger *N* = 347**	**Senior *N* = 366**	***T***	**Secure *N* = 304**	**Insecure *N* = 427**	***t***
1	Personal Costs	3.76 (1.50)	3.78 (1.43)	−0.18	4.02 (1.43)	3.41 (1.49)	5.57^***^	3.83 (1.48)	3.77 (1.48)	−0.51
2	Bureaucracy	4.11 (0.97)	4.02 (0.93)	1.33	3.99 (0.94)	4.14 (0.98)	−2.07^*^	4.03 (0.99)	4.09 (0.93)	0.81
3	Responsibility toward patients	3.25 (0.99)	3.55 (1.06)	−4.24^***^	3.43 (1.04)	3.33 (1.07)	1.19	3.24 (1.09)	3.45 (0.96)	2.78^**^
4	Role Uncertainty	4.28 (0.96)	4.50 (0.92)	−3.20^**^	4.45 (0.93)	4.26 (1.00)	2.64^**^	4.29 (0.99)	4.42 (0.91)	1.78
5	Relationship with Colleagues and staff	3.21 (0.98)	3.44 (1.07)	−3.12^**^	3.36 (1.03)	3.21 (1.04)	2.02^*^	3.17 (1.05)	3.36 (1.00)	2.48^*^
6	Global Job Satisfaction	4.55 (1.07)	4.38 (1.18)	2.05^*^	4.39 (1.11)	4.58 (1.15)	−2.24^**^	4.56 (1.11)	4.39 (1.13)	−2.09^*^

* *p < 0.05*,

** *p < 0.01*,

*** *p < 0.001*.

We divided participants into two groups on the basis of their age, using the median split method. We split the sample in two groups relating to the average of age (from 24 to 53 years and from 55 to 83 years).

Younger subjects and senior ones rated differently relating to personal costs (*p* < 0.001); Decline in Public Image and Role Uncertainty (*p* = 0.007); Bureaucracy (*p* = 0.039); Relationship with colleagues and staff (*p* = 0.044); and Global Job Satisfaction (*p* < 0.001). The younger population perceived lower levels of job satisfaction and higher levels of dissatisfaction; but they suffered less than senior to the bureaucratization. There weren't differences regarding physician's Responsibility toward patients.

One hundred and twenty-two physicians did not complete this question. 41.6% of the subjects classified themselves as secure, 13.3% as anxious, and 45.1% as avoidant.

In line with up to date literature, in our sample the attachment assessment allowed us to identified differences between secure and insecure subjects when confronted with relational issues: insecure subjects feel more uncomfortable than secure ones when charged with the responsibility of severely ill patients (*p* = 0.006), and when in trouble because of relational problems with colleagues and staff (*p* = 0.013). Moreover, insecure subjects feel less satisfied than secure physicians (*p* = 0.037). No differences emerged relating to Personal costs, Bureaucracy, and Role Uncertainty.

To examine invariance of factor structure between women and men subgroups, and between senior and young subgroups, and between secure and insecure subgroups, the multi-sample procedure was applied. In four models, we tested: (a) the configural invariance (i.e., the same number of factors across the groups; Baseline model); (b) the invariance of factor loadings (λ_x_; Model 1); (c) the invariance of error variances (θ_δ_; Model 2); (d) the invariance of correlations and variances (Φ; Model 3; see Bobbio and Manganelli, 2011). The chi-square difference test was used to compare nested models.

Fit indices and chi-square differences are summarized in Tables 4–**6**.

**Table 4:** **Fit indices and chi-square differences for the multi-sample analysis (men vs. women)**.

**Fit indices**	**Model**			
	**Baseline model**	**Model 1**	**Model 2**	**Model 3**
$\chi_{\left(\mathrm{df}\right)}^{2}$	885.21 (320)	915.28 (335)	958.81 (355)	978.07 (370)
*P*	0.00	0.00	0.00	0.00
CFI	1.00	1.00	1.00	1.00
RMSEA	0.00	0.01	0.01	0.01
SRMR	0.06	0.07	0.07	0.08
$\chi_{\Delta \left(\mathrm{df}\right)}^{2}$	−	30.07 (15)	43.53 (20)	19.26 (15)
*P*	−	0.012	0.002	0.20

*Baseline model tested configural invariance; model 1 tested invariance of factor loadings; model 2 tested invariance of error variances; model 3 tested invariance of correlations; df, degrees of freedom; CFI, Comparative Fit Index; RMSEA, Root Mean Square Error of Approximation; SRMR, Standardized Root Mean Square Residual*.

As can be seen, the invariance of factor loadings and of error variances was not confirmed for the women-men comparison. We then constrained each factor loading, one at time, to be equal across the two groups (with the exception of the first factor loading of each factor, which was fixed to one for model identification). Each model was compared to the baseline model using the chi-square difference test. Results showed significant differences across the two groups on the loadings items Item 3 (0.66 and 0.74, *ps* < 0.001 for men and women, respectively), Item 4 (0.61 and 0.72, *p*s < 0.001 for men and women, respectively), Item 11 (0.50 and 0.45, *p*s < 0.001 for men and women, respectively), Item 20 (0.35 and 0.56, *p*s < 0.001 for men and women, respectively; see Table 1). All [$\chi_{\Delta \left(1\right)}^{2}$ ≥ 3.71, *p*s ≤ 0.054].

The same procedure was applied to error variances; the model including the equality constraint was compared to the model testing the invariance of factor loadings (Model 1); significant differences were found between men and women on the error variance of items Item16 (0.84 and 0.81, for men and women, respectively, *p*s < 0.001), Item 15 (0.77 and 0.66, for men and women, respectively, *p*s < 0.001), Item 13 (0.64 and 0.63, for men and women, respectively, *p*s < 0.001), Item 19 (0.79 and 0.65, for men and women, respectively, *p*s < 0.001), all $\chi_{\Delta \left(1\right)}^{2}$ ≥ 7.12, *p*s ≤ 0.01.

Invariance of factor loadings, error variances and correlations was confirmed for the senior-young comparison (see Table 5), and for the secure-insecure comparison (see Table 6).

**Table 5:** **Fit indices and chi-square differences for the multi-sample analysis (senior vs. young)**.

**Fit indices**	**Model**			
	**Baseline model**	**Model 1**	**Model 2**	**Model 3**
$\chi_{\left(\mathrm{df}\right)}^{2}$	793.49 (320)	812.37 (335)	840.50 (355)	854.44 (370)
*P*	0.00	0.00	0.00	0.00
CFI	1.00	1.00	1.00	1.00
RMSEA	0.00	0.00	0.00	0.00
SRMR	0.06	0.06	0.07	0.07
$\chi_{\Delta \left(\mathrm{df}\right)}^{2}$	−	18.88 (15)	28.13 (20)	13.94 (15)
*p*	−	0.22	0.11	0.53

*Baseline model tested configural invariance; model 1 tested invariance of factor loadings; model 2 tested invariance of error variances; model 3 tested invariance of correlations; df, degrees of freedom; CFI, Comparative Fit Index; RMSEA, Root Mean Square Error of Approximation; SRMR, Standardized Root Mean Square Residual*.

**Table 6:** **Fit indices and chi-square differences for the multi-sample analysis (secure vs. insecure)**.

**Fit indices**	**Model**			
	**Baseline model**	**Model 1**	**Model 2**	**Model 3**
$\chi_{\left(\mathrm{df}\right)}^{2}$	818.08 (320)	830.92 (335)	853.87 (355)	876.35 (370)
*p*	0.00	0.00	0.00	0.00
CFI	1.00	1.00	1.00	1.00
RMSEA	0.00	0.00	0.00	0.00
SRMR	0.07	0.07	0.07	0.08
$\chi_{\Delta \left(\mathrm{df}\right)}^{2}$	–	12.84 (15)	22.95 (20)	22.48 (15)
*p*	–	0.61	0.29	0.10

*Baseline model tested configural invariance; model 1 tested invariance of factor loadings; model 2 tested invariance of error variances; model 3 tested invariance of correlations; df, degrees of freedom; CFI, Comparative Fit Index; RMSEA, Root Mean Square Error of Approximation; SRMR, Standardized Root Mean Square Residual*.

### Regression analysis

Multiple regression analyses were used to explore the effects of physicians' dissatisfaction on Global Job Satisfaction.

There was a constant effect of age, ranging from β = 0.12, *SE* = 0.01, *p* = 0.046, to β = 0.17, *SE* = 0.01, *p* = 0.004).

The regression analysis [*F*_(6, 732)_ = 19.63, *p* < 0.001, *R*^2^ = 0.14], showed that high levels of dissatisfaction relating to personal cost (β = −0.09, *SE* = 0.03, *p* = 0.030), bureaucracy (β = −0.19, *SE* = 0.05, *p* < 0.001), and problems with the staff (β = −0.14, *SE* = 0.05, *p* = 0.001), predict low job satisfaction.

The regression analysis showed some sex differences. Whereas males' low job satisfaction is predicted by increasing bureaucratic procedures [*F*_(6, 441)_ = 13.20, *p* < 0.001, *R*^2^ = 0.15; β = −0.27, *SE* = 0.06, *p* < 0.001]; relational problems with colleagues and staff (β = −0.24, *SE* = 0.08, *p* = 0.002), predict females' low rates of job satisfaction [*F*_(6, 271)_ = 6.77, *p* < 0.001, *R*^2^ = 0.13].

The variable which predicts low job satisfaction in insecure attached physicians is bureaucracy [*F*_(6, 361)_ = 8.39, *p* < 0.001, *R*^2^ = 0.12; β = −0.19, *SE* = 0.08, *p* = 0.006]; whereas secure attached physicians' low job satisfaction is foremost due to difficulties and relational problems with colleagues/staff (β = −0.17, *SE* = 0.08, *p* = 0.027), and bureaucracy [*F*_(6, 263)_ = 8.61, *p* < 0.001, *R*^2^ = 0.17; β = −0.19, *SE* = 0.08, *p* = 0.006]. Secure attached physicians are more sensitive to emotional and relational issues and therefore more prone to the risk of feeling uncomfortable when confronted with uncooperative staff's and colleagues' attitudes.

## Discussion

This study represents an original and innovative attempt to address physicians' dissatisfaction and job satisfaction. We developed the PhyDis Scale to measure physicians' dissatisfaction applying a mixed-method research design in order to capture the different and quickly changing sources of physicians' dissatisfaction. This study reports the development of a new measurement instrument to assess physicians' work-related stressors (PhyDis Scale), starting from their roots, that is from physicians' perception and subjective experience. We identified different, complex and probably inter-related sources of distress and dissatisfaction reported by physicians. Perceived increasing responsibility for patients, associated to augmented frequency of patients' complaints, brings physicians to pay often escalating liability insurance's costs. The almost widespread use of technology and the surge in competency maintenance requirements (Williams and Skinner, 2003; Bodenheimer and Sinsky, 2014; Schrijver, 2016) must be considered as additional stressors. The explosion of medical knowledge and of the therapeutic choices available to physicians causes an increased perception of responsibility toward patients. (Crosson and Casalino, 2015) In line with the above mentioned studies, the exploratory qualitative study and the subsequent confirmatory factor analysis of the 21 identified items of the PhyDis Scale revealed a five-dimensional factor structure: *Personal costs; Role uncertainty; Physician's Responsibility toward patients; Relationship with colleagues* and staff and *Bureaucracy*. The final version of the scale includes 20 items. Results indicated an adequate fit of the five-factor structure. The PhyDis subscales appear to be internally reliable. The external validity of the scale was confirmed, given that each source of dissatisfaction relates negatively to Job Satisfaction.

Since job satisfaction has a positive effect on patients' adherence to treatment and since it is associated with the quality of medical care, we point out that an integrated approach is crucial. The multifaceted feature of physicians' dissatisfaction has been recognized and we assume that one cannot longer rely on single-factor or even single-item scales to assess physicians' dissatisfaction. Even though international comparative studies carried out across different health care systems (Siegrist et al., 2010) reveal that effort-reward imbalance, economic issues and esteem/recognition determine a substantial part of the variation in physicians' stress levels, other studies (Quinn et al., 2009; Sinsky, 2015) identify also work-life issues and psychosocial work conditions such as esteem, social support, social conflicts with staff (Angerer and Weigl, 2015) as important sources of physicians' dissatisfaction. In line with those findings participants identified *Role Uncertainty* associated to loss of social esteem as the most dissatisfying factor. In line with up to date literature (Bovier and Perneger, 2003; French et al., 2004; Bogue et al., 2006; Davenport et al., 2008; McNearney et al., 2008; Hills et al., 2012) increasing age consistently shows to be associated with higher job satisfaction. Since attachment style is recognized as one of the most relevant variable of individual difference affecting social competences we assessed it in our convenience sample. Moreover, this is the first time attachment style's assessment in physicians is introduced in a cross-sectional study concerning the issues of *dissatisfaction* and *job satisfaction*.

According to literature on management and organizational studies (Richards and Schat, 2011) attachment insecurity predicts both job burnout (Mucci et al., 2015), and low rates of job satisfaction. Nevertheless, with the exception of Ciechanowski et al.'s (2004) study, attachment style in physicians has not yet been studied. In our study *secure* subjects were less dissatisfied than *insecure* ones, in fact, according to literature (Mikulincer and Shaver, 2007) secure subject perceive negative events as less threatening than insecure subjects. Moreover, 70% of *insecure* physicians was represented by avoidant subjects; their low job satisfaction rates were predicted by *Bureaucracy*. Avoidant subjects typically distrust others' goodwill and pursue autonomy and emotional distance from others, they can therefore rarely be affected by negative relational stances such as conflicts with colleagues and staff.

It is necessary to acknowledge some limitations of this study. The first limitation is inherent to the fact that it is based entirely on self-report measures. Secondly the exclusive reliance on cross-sectional data prevents us from making causal inferences. Even though our data support the initial validation of the multi-factorial scale, future research should compare physicians' dissatisfaction rates from the three major different systems of health care provision: private insurance-based system (US); government-supported, tax-based system (UK); and mixed system (Europe) (Siegrist et al., 2010); a comparison should also be made between countries with low/middle incomes and those with high incomes. Further limitations refer to the fact that participants are employed in a mixed health system and future research should be devoted to identify differences in the variety and range of dissatisfaction sources between private and mixed systems. Item's wording could not be pre-tested in order reduce possible slight differences in the understanding of items' meaning. Future longitudinal studies could explore the variability of dissatisfaction sources over time. Last but not least, further exploration is needed in the testing of the model within the women and men subgroups.

## Conclusion

The final version of the PhyDis scale includes 20 items. Results indicated an adequate fit of the five-factor structure. The PhyDis subscales appear to be internally reliable. The external validity of the scale was confirmed, given that each source of dissatisfaction relates negatively to Job Satisfaction.

Invariance of correlations was confirmed for each considered subgroup: for the senior-young, the women-men and for the insecure-secure attached subgroups. The invariance of factor loadings and error variance was confirmed for the senior-young and for the insecure-secure attached populations. These last were not confirmed for the women-men groups.

*Role Uncertainty* associated to loss of social esteem is the most dissatisfying factor. Increasing age consistently shows to be associated with higher job satisfaction and with higher dissatisfaction relating to *Bureaucracy*.

Attachment style is recognized as important variable of individual difference affecting social competence and coping strategies in interpersonal relational contexts, with special regard to health/and helping-professions' organizations. For the first time attachment style's assessment in physicians is introduced in a cross-sectional study concerning the issues of *dissatisfaction* and *job satisfaction*. The insecure sub-sample (58.4%) of our population is represented by physicians, whose low job satisfaction rates are predicted by *Bureaucracy*. By contrast secure attached physicians' (41.6%) low job satisfaction is paramount due to difficulties in relational problems with staff and colleagues.

Whilst addressing factors that may trigger dissatisfaction such as collegial support and work demands may have a tangible positive effect on the quality of care, physicians' attachment style assessment may be useful to offer appropriate and differentiated training opportunities to secure and insecure healthcare professionals allowing them to contribute to improving the quality of care. In addition lower levels of dissatisfaction due to *Bureaucracy* in the younger physicians' population could be associated to their higher computer literacy which allows them to cope easily with various tasks connected to the CPR (computer-based patient record). Whereas the older population could benefit from computer-training (Rahimi et al., 2008), the younger sub-group could benefit from conflict-resolution training courses in order to increase the number of coping strategies they rely on managing the physician/patient and the physician/staff relationships.

## Author contributions

All authors listed, have made substantial, direct and intellectual contribution to the work, and approved it for publication.

## Funding

The authors received no financial support for the research, authorship, and/or publication of this article.

### Conflict of interest statement

The authors declare that the research was conducted in the absence of any commercial or financial relationships that could be construed as a potential conflict of interest.
